# The relationship between breast milk intake and speech in children with cerebral palsy

**DOI:** 10.3906/sag-2011-43

**Published:** 2021-08-30

**Authors:** Gül Demet KAYA ÖZÇORA, Gonca BEKTAŞ

**Affiliations:** 1 Department of Pediatric Neurology, Faculty of Health Sciences, Hasan Kalyoncu University, Gaziantep Turkey; 2 Department of Pediatric Neurology, Bakırköy Dr. Sadi Konuk Research and Training Hospital, İstanbul Turkey

**Keywords:** Cerebral palsy, breast milk, speech disorders

## Abstract

**Background/aim:**

Cerebral palsy (CP) is a nonprogressive neurodevelopmental disorder that cause damage to the developing brain (0–3 years) for various reasons. Children with CP commonly have speech disorders due to impairment in neuromuscular control of oro-motor coordination. We focused on the relationship between breast milk intake and speech functions in children with CP.

**Materials and methods:**

The gross motor function classification system (GMFCS) was used to categorize the gross motor function. The viking speech scale (VSS) was used to classify the speech in children with cerebral palsy. Children were subdivided into two groups as term and preterm based on gestational age. The duration of exclusive breast milk intake was defined as the period when the infant received breast milk alone. We used Spearman’s correlation coefficient to evaluate the relationship between the duration of breast milk intake, GMFCS, and VSS.

**Results:**

The median level of viking speech scale was 2 in preterm-born children and 4 in term-born children. There was no correlation between age and VSS levels.We observed a statistically significant difference in terms of preterm- or term-born status among children with different VSS levels. There was a weak positive correlation between birth weight and VSS level, indicating better speech function in children with lower birth weight. There was a moderate negative correlation between the duration of exclusive breast milk intake, the total duration of breast milk intake, and the corrected age of weaning completion with VSS level.

**Conclusion:**

The duration of breast milk intake may reflect the oromotor function and predict speech performance in children with cerebral palsy. We wanted to emphasize that speech language therapy is as important as motor rehabilitation.

## 1. Introduction

Cerebral palsy (CP) is a nonprogressive neurodevelopmental -disorder that cause damage to the developing brain (03 y) for various reasons, and causes loss of motor function, movement- and posture. It is the most common cause of motor impairment in children [1]. The prevalence of CP has increased in the last 40 years, reaching 2 per 1000 live births and may develop due to many factors in prenatal, natal or early postnatal period [13].

-Although CP ‘s main symptom is the loss of motor function ,it is often accompained by epileptic, auditory, visual,cognitive,perceptive and behavioral disorders. These accompanying disorders sometimes affect the patients’ daily life functions and quality of life more than the motor dysfunction. Children with CP commonly have speech disorders due to impairment in neuromuscular control of oro-motor coordination [2,4]. of communication has significantİmpairment adverse effects on social participation, educational participation and overall quality of life.

There are a few studies addressing speech and language interventions in children with cerebral palsy [5]. The positive influence of breast milk intake on brain maturation has widely been reported in healthy children [68]. Breastfeeding and breast milk intake may induce neuroplasticity in- the early period of life in children with cerebral palsy and improve neurologic outcomes. Therefore, we focused on the relationship between breast milk intake and speech functions in children with cerebral palsy.

## 2. Materials and methods

This cross-sectional, observational study consisted of children with cerebral palsy who were under follow-up of pediatric neurology outpatient clinics from March 1, 2019, to July 31, 2019. Children with cerebral palsy with aged between 4 and 18 years and admitted to the outpatient clinic for regular follow-up during the study period were included in the study. Premature and term patients with similar age and gross motor function classification system (GMFCS) level distribution were included. The exclusion criteria were as follows; presence of hearing loss, orofacial cleft, inherited metabolic disorders affecting the central nervous system, the presence of progressive disease signs, dysmorphic features, moderate-severe mental impairment, iron deficiency anemia, vitamin b12 deficiency, the presence of refracter epilepsy and insufficient data. The ethics committee approved the study protocol. All participants agreed to participate in the study and the guardian of each subject signed a written informed consent form.

### 2.1.Clinical ata

**D**Clinical data regarding demographic features, birth weight, gestational age at the time of delivery and length of stay in the neonatal intensive care unit (NICU) were extracted from medical records. Mothers were asked a set of questions including the number of siblings, the duration of exclusive breast milk intake, the total duration of breast milk intake and the corrected age of weaning completion at last follow-up or via phone call. Children were subdivided into two groups as term (born at > 37 weeks) and preterm (born at ≤ 37 weeks) based on gestational age. The duration of exclusive breast milk intake was defined as the period when the infant received breast milk alone.

### 2.2.Classification of motor function and speech

The gross motor function classification system (GMFCS) was used to categorize the gross motor function into five levels, in which higher levels indicate a higher degree of motor disability [9,10]. The Turkish version of expanded and revised GMFCS was defined as a reliable and valid tool for children with cerebral palsy aged up to 18 years [11]. The viking speech scale (VSS) was used to classify the speech in children with cerebral palsy. It was reported that VSS was a reliable and valid tool in children with cerebral palsy aged over 4 years. The VSS categories range from level I to level IV, in which level I indicates no impairment of speech, and level IV indicates no understandable speech [12,13].

### 2.3.Statistical analysis

All statistical analyses were performed using the SPSS Statistics for Windows 23.0 software package (IBM Corp., Armonk, NY,version USA). The KolmogorovSmirnov test was used to determine whether variables were normally distributed or not. Demographic and clinical variables were compared between different VSS levels using analysis of variance (ANOVA) with Bonferroni corrections in the case of normality or otherwise with the Kruskal-Wallis test. We used Spearman’s correlation coefficient to evaluate the relationship -between the duration of breast milk intake, GMFCS and VSS. Binary logistic regression analysis was performed on variables with an unadjusted effect. The VSS levels were subdivided into two categories: good function (level I or II) and poor function (level III or IV) to perform logistic regression analysis. A value of p < 0.05 was accepted as statistically significant.

## 3.Results

3.1.Participants descriptive characteristics

A total of 251 (129 premature, 122 mature) children with cerebral palsy GMFSC level 123 were included in the study. The mean age of children was 7.3 (standard deviation [SD] 3.3; range, 417) years. The male (n-=153, 61%) to female (n=98, 39%) ratio was 1.6. The mean birth weight was 2346 (SD 1067;grams range, 6504750) Overall, 129 children (51%) were born preterm and 122 children (49%) were born at term. The mean sibling count was 454 (SD 2.05; range,09). The mean length of stay in the NICU was 31.4 (SD 39.8; range, 1270) days.

,-The mean duration of exclusive breast milk intake was 56.9 (SD 65.2 days; range, 0180) days. The mean duration of -total breast milk intake was 7.4 (SD 8.2; range, 036) months. Forty-three children (17%) received no breast milk. The demographic and clinical variables are presented in Table 1. The median level of VSS was 2 (range, 14) in preterm-born children and 4 (range, 1-4) in term-born children. 

**Table 1:** Demographic and clinical variables of children with cerebral palsy.

	Children with cerebral palsy
n	251
Male, Female ,n (%)	153 (61%), 98 (39%)
The mean age (year)	7.3 (4–17)
The mean birth weight (g)	2346 (650–4750)
Born preterm, n (%)	129 (51%)
Born term, n (%)	122 (49%)
The mean length of stay in the NICU ( day)	31.4 (0–270)
The mean duration of exclusive breast milk intake (day)	56.9 (0–180)
The mean duration of total breast milk intake (month)	7.4 (0–36)
Breastfeeding, n (%)	142 (57%)
Bottle feeding, n (%)	66 (26%)
No breast milk, n (%)	43 (17%)
The level of VSS-Level I, n (%)	35 (14%)
The level of VSS-Level II, n (%)	59 (24%)
The level of VSS-Level III, n (%)	46 (18%)
The level of VSS-Level IV, n (%)	111 (44%)

## 3.2.The association of speech with other variables

There was no statistically significant difference regarding between the different VSS genderlevels (p= 0.29). There was no correlation between age and VSS levels (p= 0.79). We observed a statistically significant difference in terms of preterm- or term-born status among children with different VSS levels (p= 0.001). The VSS levels and born status are presented in Table 2. There was a weak positive correlation between birth weight and VSS level, indicating better speech function in children with lower birth weight (p= 0.02, r= 0.28). There was no correlation between the length of stay in the NICU and the VSS level (p= 0.19). There was no correlation between the sibling count and the VSS level (p= 0.54). There was a moderate negative correlation between the duration of exclusive breast milk intake, the total duration of breast milk intake, and the corrected age of weaning completion with VSS level (p<0.001, r= 0.39, p=0.001, r= 0.36, respectively) (Figure)

**Table 2:** The VSS levels and born status.

VSS	Preterm (N/%)	Term (N/%)
Level I, n (%)	29 (83%)	6 (17%)
Level II, n (%)	37 (62%)	22 (38%)
Level III, n (%)	26 (56%)	20 (44%)
Level IV, n (%)	38 (34%)	73 (65%)

**Figure 1:**
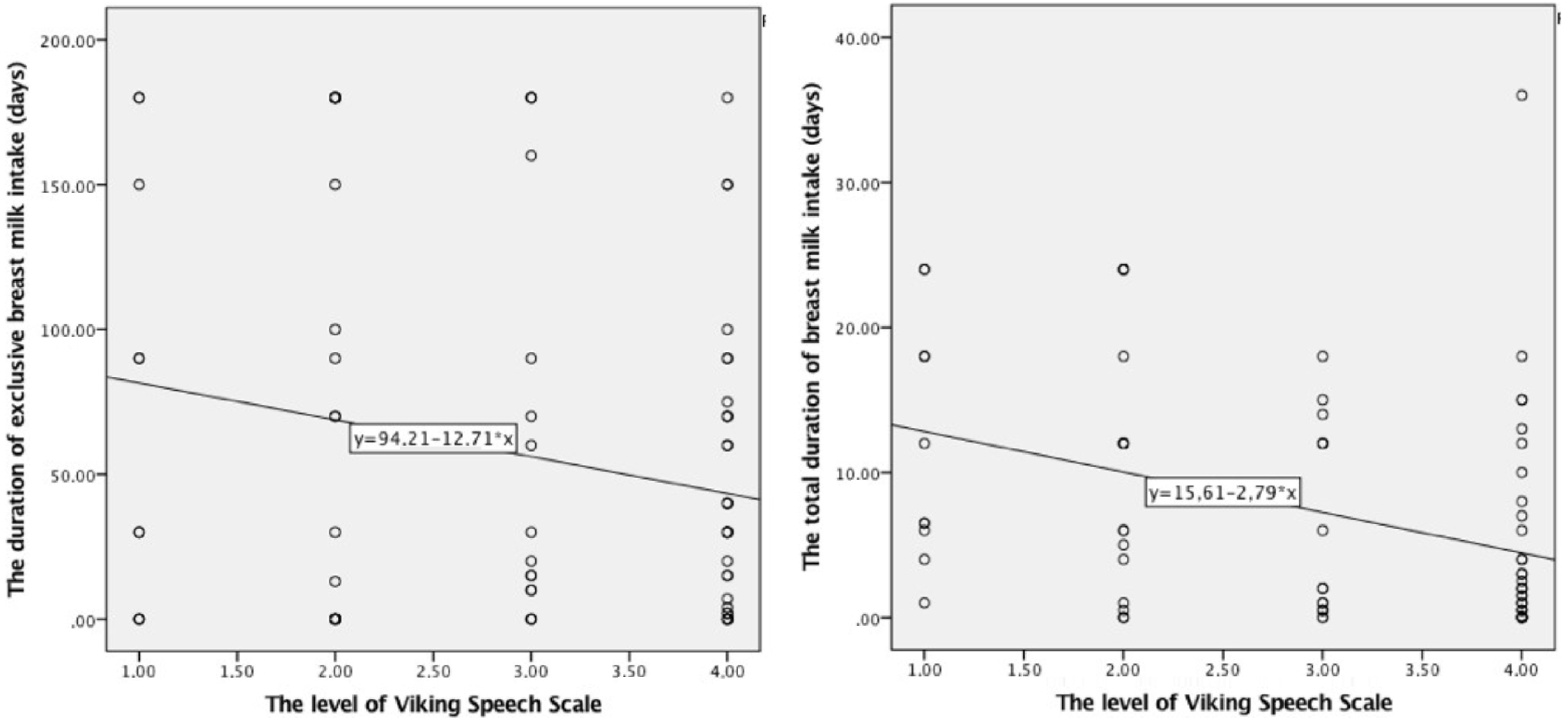
Correlation between the levels of speech classified by the viking speech scale and the duration of exclusive breast milk intake and the total duration of breast milk intake. A negative correlation was found in both durations of breast milk intake.

## 4.Discussion

We assessed the relationship between breast milk intake and other clinical variables with speech functions in children with cerebral palsy. Children with prolonged duration of breast milk intake and children breastfed irrespective of duration were more prone to exhibiting better speech functions. This is in line with prior studies showing that breast milk intake accelerated the maturation of the frontal and temporal white matter regions associated with auditory and language areas [5].

There was a moderate negative correlation between the total duration of exclusive breast milk intake, the total duration of breast milk intake, and the corrected age of weaning completion with VSS level . Breast milk contains docosahexaenoic acid, arachidonic acid and cholesterol in contrast to formula milk. These constituents play a significant role in white matter development and myelination [6,14]. It was reported that, besides breast milk intake, the duration of breast milk intake was also positively correlated with verbal functions and the cortical thickness of the parietal lobe [7].

Breastfeeding may improve speech by inducing the plasticity of brain regions associated with oral motor and articulatory control in the early period of life in children with cerebral palsy [15]. It has been suggested that early interventions and integration of these interventions requiring active participation in daily life activities were more likely to improve neurologic outcomes in children with cerebral palsy [1517]. -From this perspective breastfeeding may be an efficient early intervention for achieving oral motor and articulatory control.

There was no statistically significant difference observed regarding between the different VSS levels (p= 0.29). gender We observed a statistically significant difference in terms of preterm- or term-born status among children with different VSS levels.There was a weak positive correlation between birth weight and VSS level, indicating better speech function in children with lower birth weight (p= 0.02, r= 0.28). Children born preterm and consequently with low birth weight presented better speech functions than children born at term and with higher birth weight. It has been proposed that the brain regions’ vulnerability varies depending on the timing of the lesion. The lesions that emerge in the cortical-subcortical regions at term age may express more severe dysfunction than the lesions that develop in the periventricular white matter at preterm age [16]. This could explain why children born preterm had better speech function.

There was no correlation between the length of stay in the NICU and the VSS level (p =0.19). Forty-three children (17%) received no breast milk. Regardless of the length of stay in the NICU leads to a reduction in the rate of breastfeeding and duration of breastfeeding. We think that mother- baby contact should be accomplished as soon and much as possible. 62% of patients have advanced VSS level (34), although advanced GMFCS levels were not included the study.-This situation may be due to the lack of attention importance of language therapy and bilingualism at home. Bilingualism was detected in 39% of these cases and29% of all cases. Our studys’ strengths include its large sample size and study design, which allowed usto identify the influence of variables on speech via a reliable and valid classification system. The study limitations are the etiological and radiological classification could not be done.

The duration of breast milk intake may reflect the oromotor function and predict speech performance in children with cerebral palsy. We wanted to emphasize that speech language therapy is as important as motor rehabilitation. We recommend monolingualism to support language development in patients with cerebral palsy.
